# Supplementary material for: A Markov random field model for network-based differential expression analysis of single-cell RNA-seq data
Source: BMC Bioinformatics. 2021 Oct 26;22:524. doi: 10.1186/s12859-021-04412-0 (PMC8549347; doi:10.1186/s12859-021-04412-0)
Supplement: Supplementary file 1 — Additional file 1. Derivation on the Markov Random Field model. [file 12859_2021_4412_MOESM1_ESM.pdf]

Additional File 2 - Proof

The details on the derivation of the conditional probability from the joint probability can be shown as

$$\begin{aligned}
&\frac{p\left(w_{gc}=1 \,|\, \mathbf{W}\setminus w_{gc}; \boldsymbol{\Phi}\right)}{p\left(w_{gc}=0 \,|\, \mathbf{W}\setminus w_{gc}; \boldsymbol{\Phi}\right)} \\
=\;& \exp\Big\{\gamma_1-\gamma_0+\beta_{\text{gene}}\sum_{g'\neq g}\Big[\mathcal{I}_1\left(w_{g'c}\right)-\mathcal{I}_0\left(w_{g'c}\right)\Big]+\beta_{\text{cell}}\sum_{c'\neq c}\Big[\mathcal{I}_1\left(w_{gc'}\right)-\mathcal{I}_0\left(w_{gc'}\right)\Big]\Big\} \\
=\;& \exp\Big\{\gamma+\beta_{\text{gene}}\sum_{g'\neq g}\left(2w_{g'c}-1\right)+\beta_{\text{cell}}\sum_{c'\neq c}\left(2w_{gc'}-1\right)\Big\} \\
=\;& \exp\big\{F\left(w_{gc},\boldsymbol{\Phi}\right)\big\}
\end{aligned}$$

where $\gamma=\gamma_1-\gamma_0$ and $F\left(w_{gc},\boldsymbol{\Phi}\right)=\gamma+\beta_{\text{gene}}\sum_{g'\neq g}\left(2w_{g'c}-1\right)+\beta_{\text{cell}}\sum_{c'\neq c}\left(2w_{gc'}-1\right)$. We have

$$p\left(w_{gc}=0 \,|\, \mathbf{W}\setminus w_{gc}; \boldsymbol{\Phi}\right)=\frac{p\left(w_{gc}=1 \,|\, \mathbf{W}\setminus w_{gc}; \boldsymbol{\Phi}\right)}{\exp\big\{F\left(w_{gc},\boldsymbol{\Phi}\right)\big\}}$$

In addition, we know that

$$p\left(w_{gc}=1 \,|\, \mathbf{W}\setminus w_{gc}; \boldsymbol{\Phi}\right)+p\left(w_{gc}=0 \,|\, \mathbf{W}\setminus w_{gc}; \boldsymbol{\Phi}\right)=1$$

It is trivial to show that

$$\begin{aligned}
p\left(w_{gc}=1 \,|\, \mathbf{W}\setminus w_{gc}; \boldsymbol{\Phi}\right)+\frac{p\left(w_{gc}=1 \,|\, \mathbf{W}\setminus w_{gc}; \boldsymbol{\Phi}\right)}{\exp\big\{F\left(w_{gc},\boldsymbol{\Phi}\right)\big\}} &= 1 \\
p\left(w_{gc}=1 \,|\, \mathbf{W}\setminus w_{gc}; \boldsymbol{\Phi}\right)\cdot\left(1+\frac{1}{\exp\big\{F\left(w_{gc},\boldsymbol{\Phi}\right)\big\}}\right) &= 1 \\
p\left(w_{gc}=1 \,|\, \mathbf{W}\setminus w_{gc}; \boldsymbol{\Phi}\right)\cdot\left(\frac{1+\exp\big\{F\left(w_{gc},\boldsymbol{\Phi}\right)\big\}}{\exp\big\{F\left(w_{gc},\boldsymbol{\Phi}\right)\big\}}\right) &= 1
\end{aligned}$$

Thus, we have

$$p\left(w_{gc}=1 \,|\, \mathbf{W}\setminus w_{gc}; \boldsymbol{\Phi}\right)=\frac{\exp\big\{F\left(w_{gc},\boldsymbol{\Phi}\right)\big\}}{1+\exp\big\{F\left(w_{gc},\boldsymbol{\Phi}\right)\big\}}$$

and

$$p\left(w_{gc}=0 \,|\, \mathbf{W}\setminus w_{gc}; \boldsymbol{\Phi}\right)=\frac{1}{1+\exp\big\{F\left(w_{gc},\boldsymbol{\Phi}\right)\big\}}$$

By combining the two cases, we have

$$p\left(w_{gc} \,|\, \mathbf{W}\setminus w_{gc}; \boldsymbol{\Phi}\right)=\frac{\exp\left\{w_{gc}\,F\left(w_{gc},\boldsymbol{\Phi}\right)\right\}}{1+\exp\left\{F\left(w_{gc},\boldsymbol{\Phi}\right)\right\}}$$
