# Supplementary material for: A Markov random field model for network-based differential expression analysis of single-cell RNA-seq data
Source: BMC Bioinformatics. 2021 Oct 26;22:524. doi: 10.1186/s12859-021-04412-0 (PMC8549347; doi:10.1186/s12859-021-04412-0)
Supplement: Supplementary file 2 — Additional file 2. Supplementary Tables and Figures. [file 12859_2021_4412_MOESM2_ESM.pdf]

Supplementary Figures and Tables of
“A Markov Random Field Model for Network-based Differential Expression Analysis of Single-cell RNA-seq Data”

Hongyu Li[1], Biqing Zhu[2], Zhichao Xu[1], Taylor Adams[3], Naftali Kaminski[3], Hongyu Zhao[1]

[1] *Department of Biostatistics, Yale School of Public Health, New Haven, CT 06511, USA*

[2] *Program of Computational Biology and Bioinformatics, Yale University, New Haven, CT 06511, USA*

[3] *Section of Pulmonary, Critical Care and Sleep Medicine, Department of Internal Medicine, Yale School of Medicine, New Haven, CT 06520, USA*

(A)

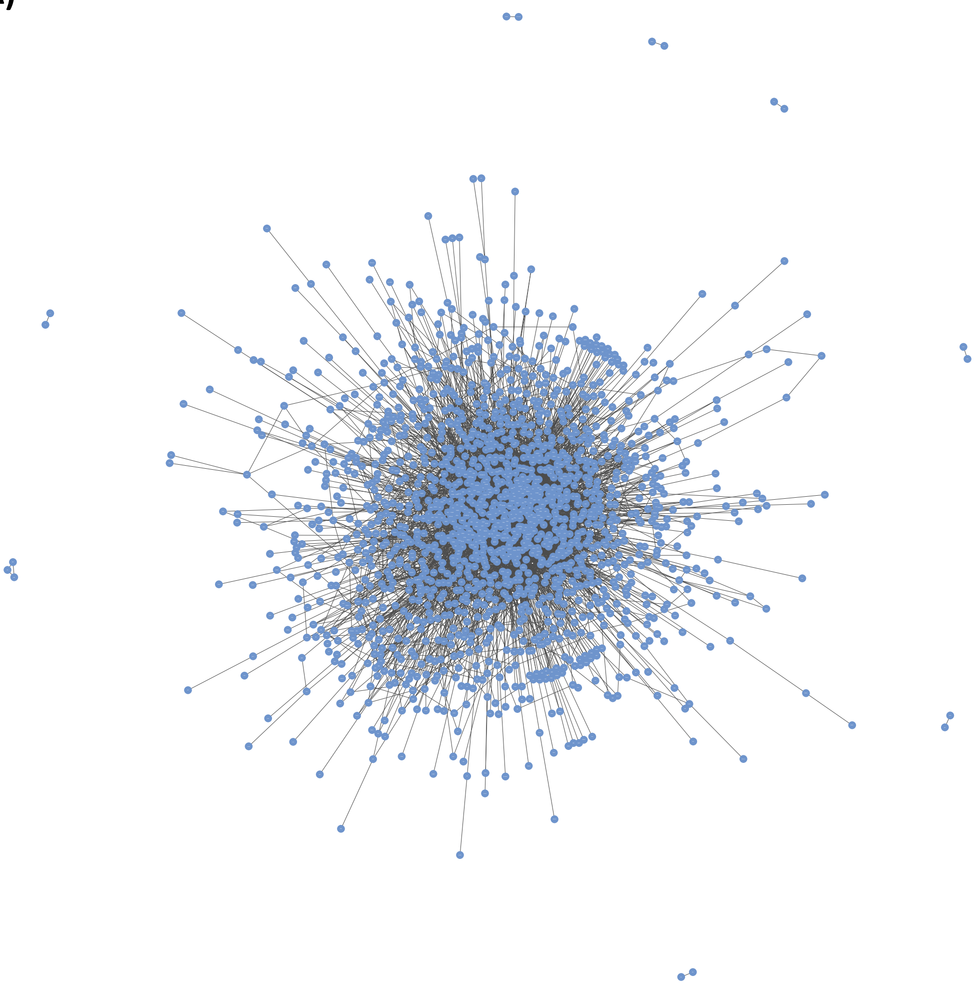

(B)

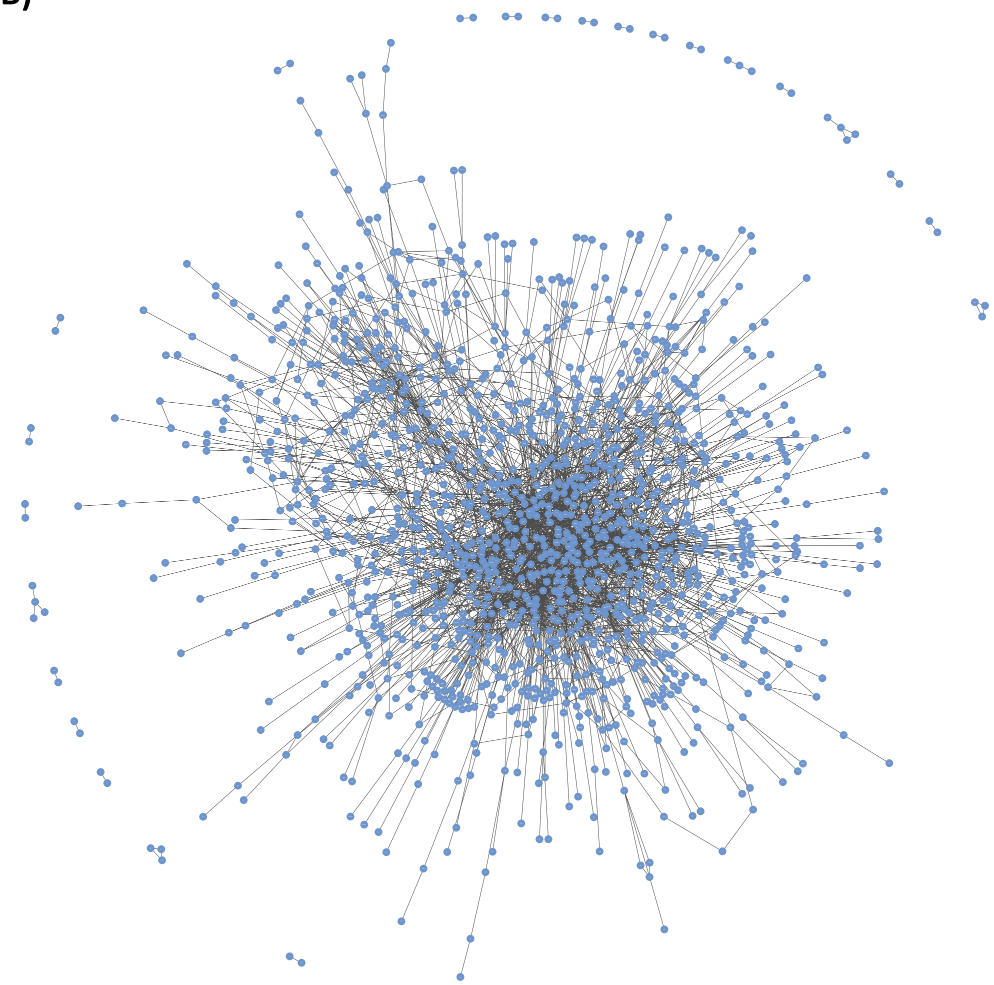

Supplementary Figure 1. (A) shows the 2,000 gene network from the BioGrid database and **(B)** shows the 2,000 gene network from the IntAct database. Each node represents a gene and each edge represents a direct relationship (interaction) between the two genes. The edges are directionless. There are 5,400 edges in **(A)** and 3,104 edges in **(B)**. There is an overlap of 1,754 edges between the two databases.

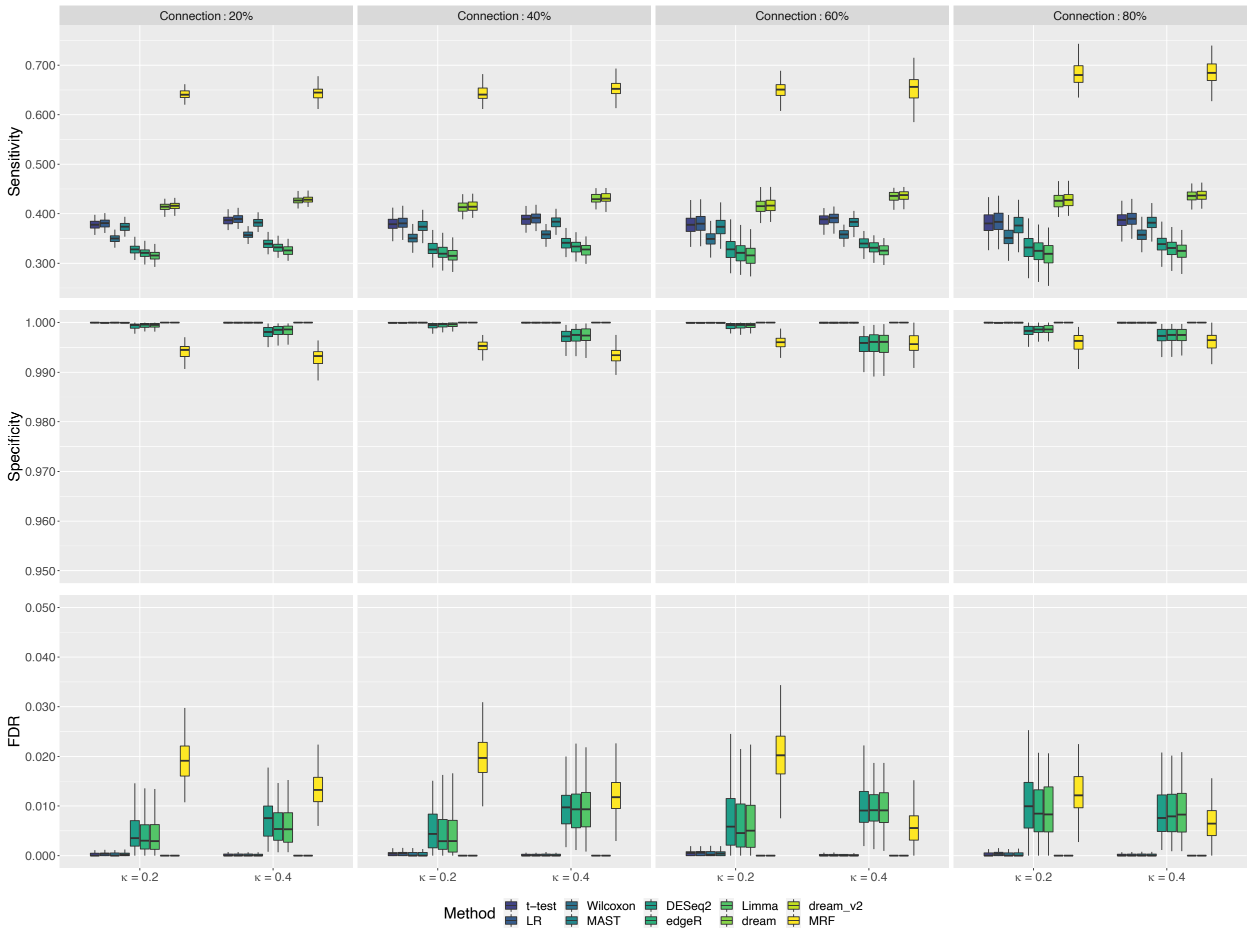

Supplementary Figure 2A. Simulation results for Scenario A. We varied the number of subjects in each group (case/control) to be 30. The results are plotted in terms of sensitivity, specificity and FDR for two-sample t-test, the likelihood ratio test that adopts a logistic regression framework, Wilcoxon rank sum test, MAST, three pseudo-bulk methods: DESeq2, edgeR, and limma, two mixed model methods: dream and its updated version dream2, and the proposed MRF model. Each box-plot represents 100 replications.

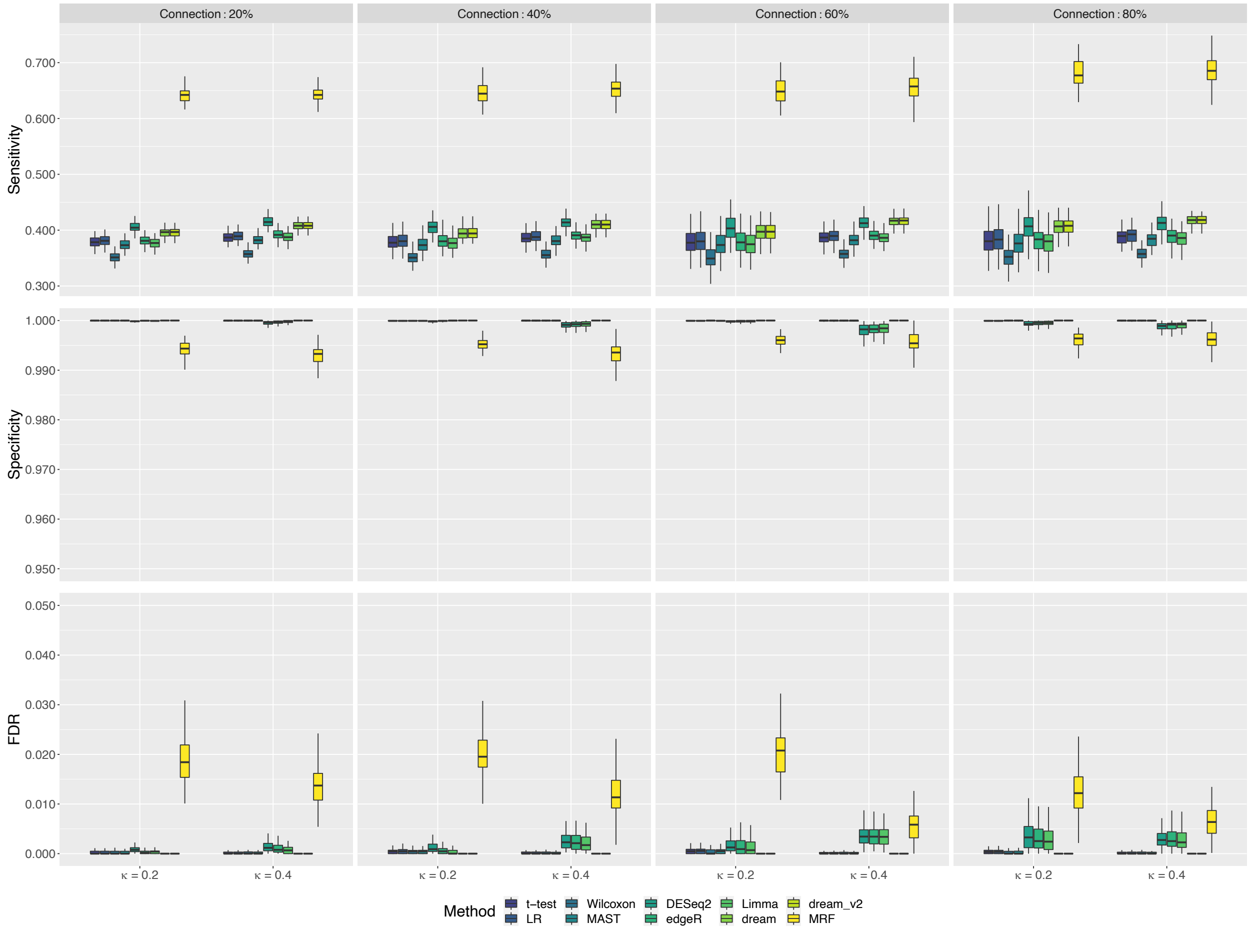

Supplementary Figure 2B. Simulation results for Scenario B. We varied the number of cells per cell type in each subject to be 100. The results are plotted in terms of sensitivity, specificity and FDR for two-sample t-test, the likelihood ratio test that adopts a logistic regression framework, Wilcoxon rank sum test, MAST, three pseudo-bulk methods: DESeq2, edgeR, and limma, two mixed model methods: dream and its updated version dream2, and the proposed MRF model. Each box-plot represents 100 replications.

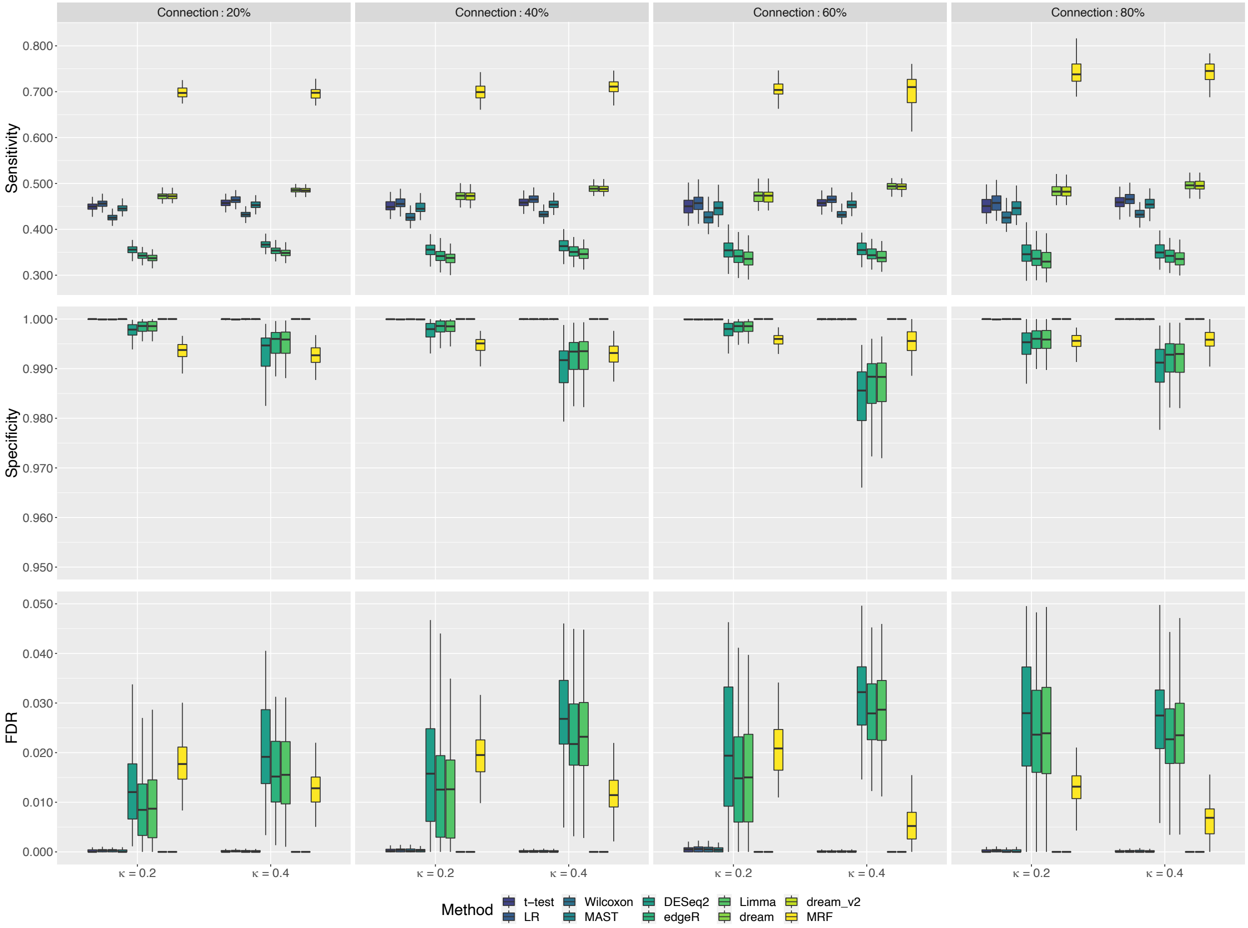

Supplementary Figure 2C. Simulation results for Scenario C. We varied the λ to be 3. The results are plotted in terms of sensitivity, specificity and FDR for two-sample t-test, the likelihood ratio test that adopts a logistic regression framework, Wilcoxon rank sum test, MAST, three pseudo-bulk methods: DESeq2, edgeR, and limma, two mixed model methods: dream and its updated version dream2, and the proposed MRF model. Each box-plot represents 100 replications.

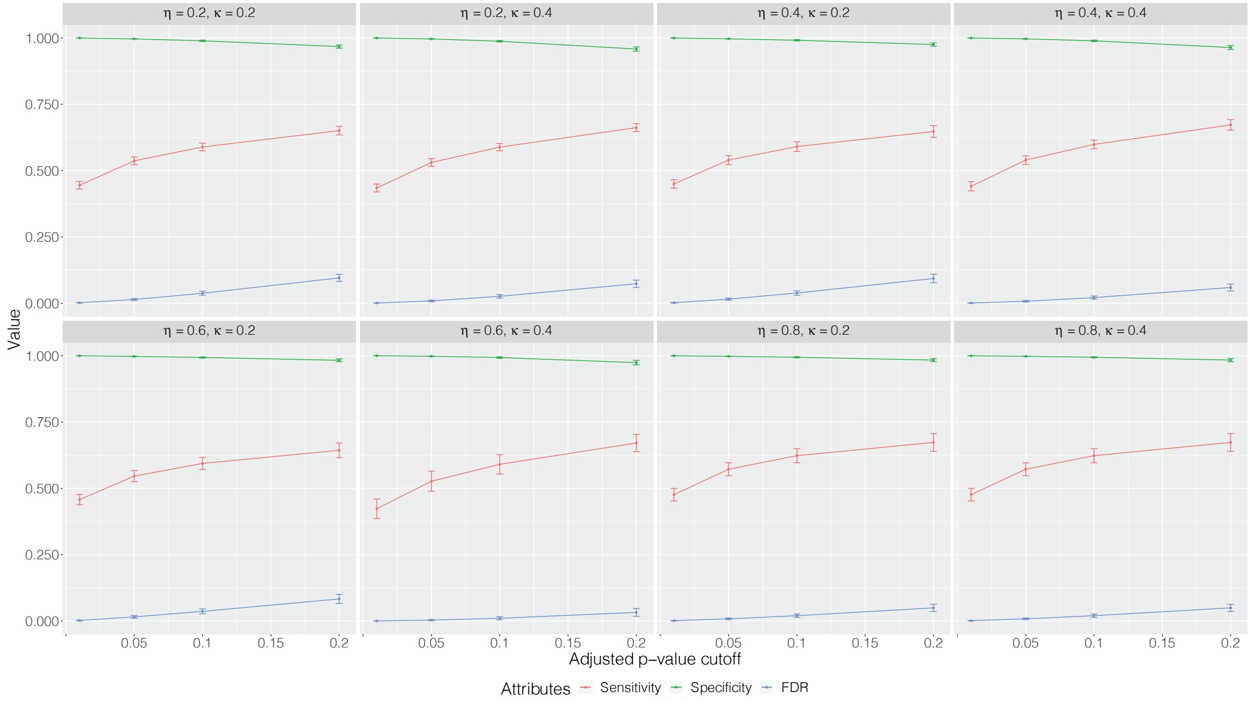

Supplementary Figure 2D. The impact of different adjusted p-value thresholds on sensitivity, specificity, and FDR. Four adjusted p-value cutoffs were used: 0.01, 0.05, 0.1, and 0.2. Sensitivity, specificity, and FDR are plotted for the proposed MRF model under different simulation settings (corresponding to the eight cases in Figure 2). When the adjusted p-value threshold increases, sensitivity increases and specificity decreases for all eight cases. Our proposed MRF model achieves the desired FDR control.

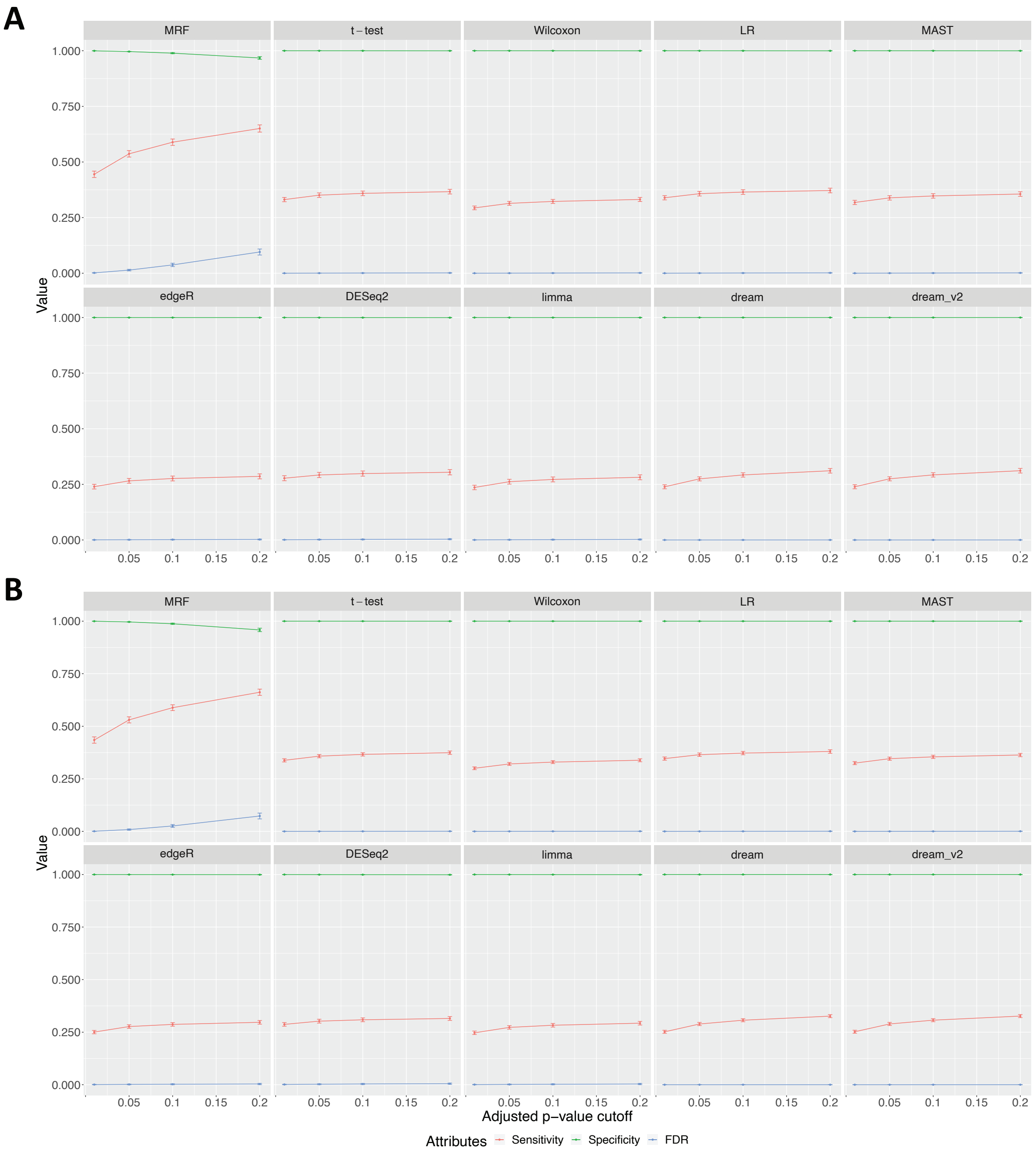

Supplementary Figure 2E. The impact of different adjusted p-value thresholds on sensitivity, specificity, and FDR. Four adjusted p-value cutoffs were used: 0.01, 0.05, 0.1, and 0.2. Sensitivity, specificity, and FDR are plotted for all ten methods under the simulation settings where $\eta = 0.2$, and $\kappa = 0.2$ or $\kappa = 0.4$ in the main manuscript (corresponding to the first two cases in Figure 2).

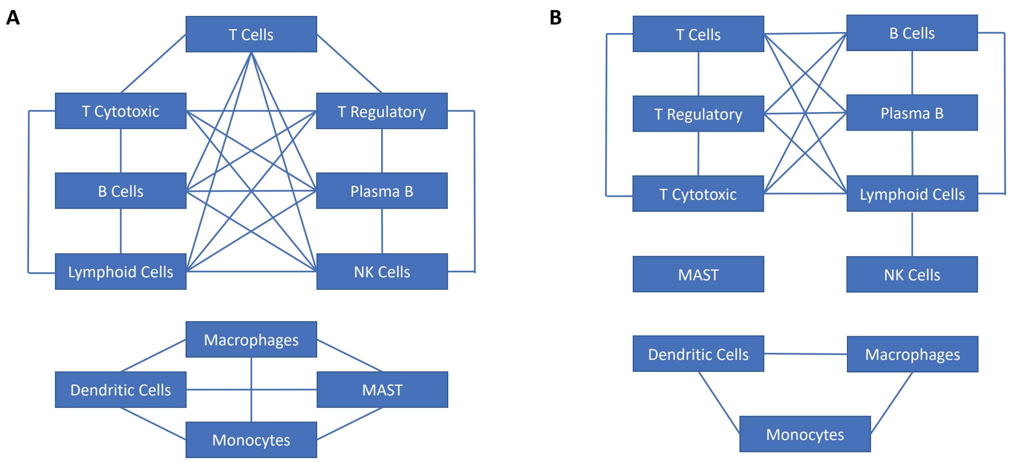

Supplementary Figure 3. The two additional cell networks we used in our analysis in order to assess the importance of cell network structures in the proposed MRF model. Models with cell network in (A) are labeled with C1, and models with cell network in (B) are labeled with C2.

Supplementary Table 1. A Summary Description of Models

Model Labels	MRF Model?	Description
**Main Models**		
Main	No	The DE results are based on two-sample t-test.
Main w/ BioGrid	Yes	It uses test statistics from two-sample t-test as DE evidence, gene network from BioGrid database, and cell network in Figure 1 to build the MRF model.
Main w/ IntAct	Yes	It uses test statistics from two-sample t-test as DE evidence, gene network from IntAct database, and cell network in Figure 1 to build the MRF model.
**Supplementary Models A**		
Wilcoxon	No	The DE results are based on Wilcoxon test.
Wilcoxon w/ BioGrid	Yes	It uses test statistics from Wilcoxon test as DE evidence, gene network from BioGrid database, and cell network in Figure 1 to build the MRF model.
Wilcoxon w/ IntAct	Yes	It uses test statistics from Wilcoxon test as DE evidence, gene network from IntAct database, and cell network in Figure 1 to build the MRF model.
MAST	No	The DE results are based on MAST analysis.
MAST w/ BioGrid	Yes	It uses test statistics from MAST analysis as DE evidence, gene network from BioGrid database, and cell network in Figure 1 to build the MRF model.
MAST w/ IntAct	Yes	It uses test statistics from MAST analysis as DE evidence, gene network from IntAct database, and cell network in Figure 1 to build the MRF model.
**Supplementary Models B**		
Main w/ BioGrid C1	Yes	It uses test statistics from two-sample t-test as DE evidence, gene network from BioGrid database, and cell network C1 in Supplementary Figure 3 to build the MRF model.
Main w/ IntAct C1	Yes	It uses test statistics from two-sample t-test as DE evidence, gene network from IntAct database, and cell network C1 in Supplementary Figure 3 to build the MRF model.
Main w/ BioGrid C2	Yes	It uses test statistics from two-sample t-test as DE evidence, gene network from BioGrid database, and cell network C2 in Supplementary Figure 3 to build the MRF model.
Main w/ IntAct C2	Yes	It uses test statistics from two-sample t-test as DE evidence, gene network from IntAct database, and cell network C2 in Supplementary Figure 3 to build the MRF model.

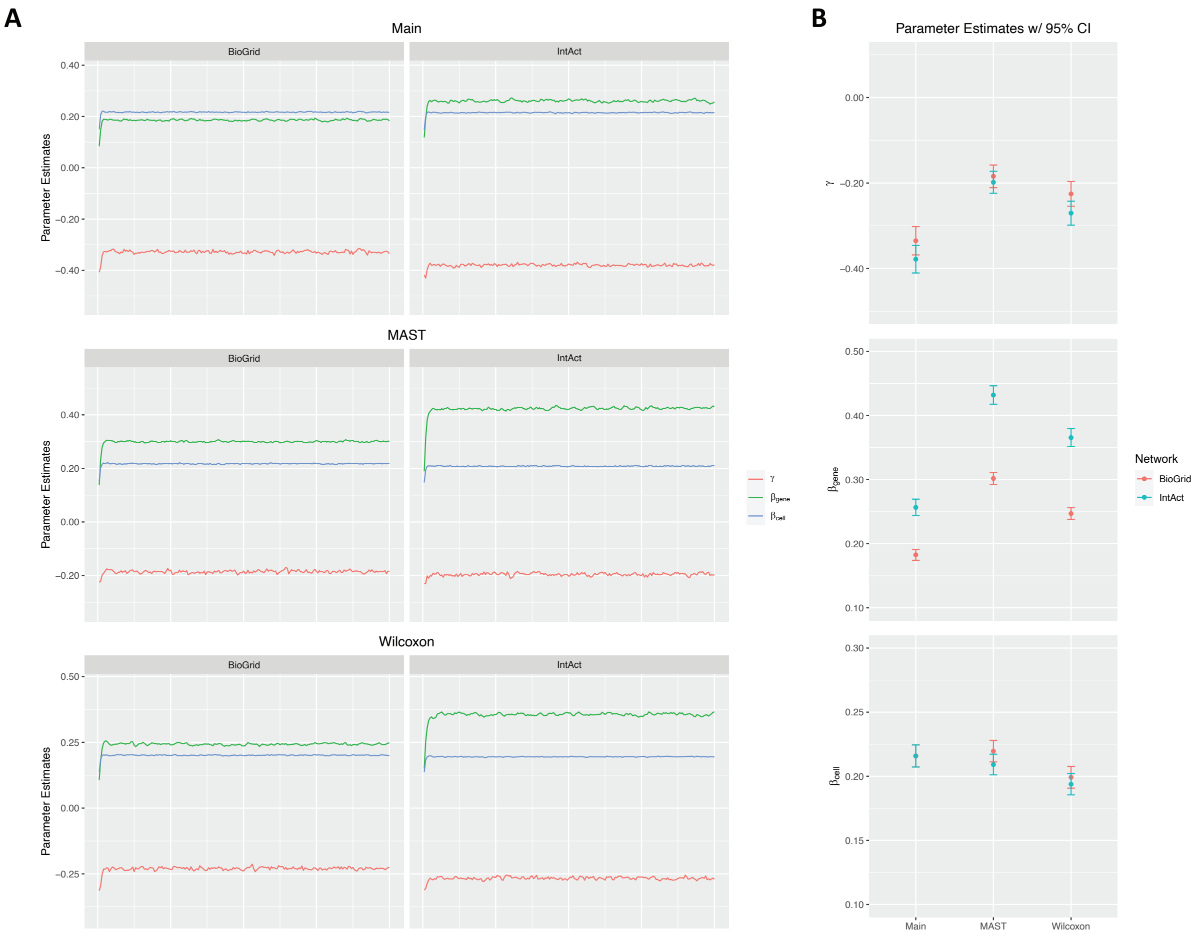

Supplementary Figure 4. The parameter estimates for the Main MRF models, MAST MRF models, and Wilcoxon MRF models with BioGrid and IntAct gene networks**. (A)** shows the trace plots for γ, β_{gene}, and β_{cell} from the EM algorithm with mean filed-like approximation. **(B)** shows the parameter estimates for γ, β_{gene}, and β_{cell} and their corresponding 95% confidence intervals.

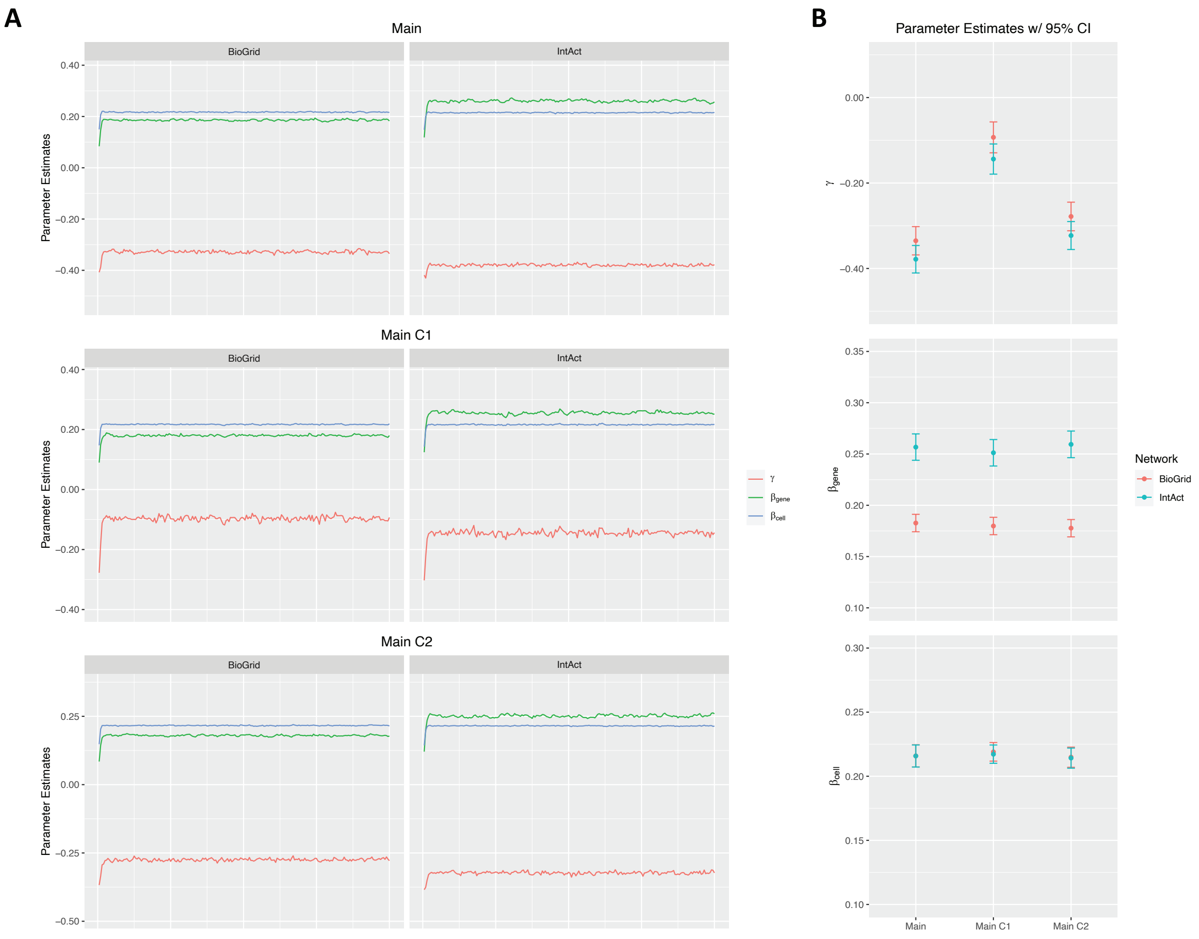

Supplementary Figure 5. The parameter estimates for the Main MRF models and the MRF models with two additional cell networks (C1 and C2) we considered. The upper panel in **(A)** shows the trace plots for γ, β_{gene}, and β_{cell} from the EM algorithm with mean filed-like approximation for the Main MRF model with BioGrid and IntAct gene networks; the middle panel in **(A)** shows the trace plots for the Main MRF model with C1 cell network, and BioGrid and IntAct gene networks; and the lower panel in **(A)** shows the trace plots for the Main MRF model with C2 cell network, and BioGrid and IntAct gene networks. **(B)** shows the parameter estimates for γ, β_{gene}, and β_{cell} and their corresponding 95% confidence intervals.

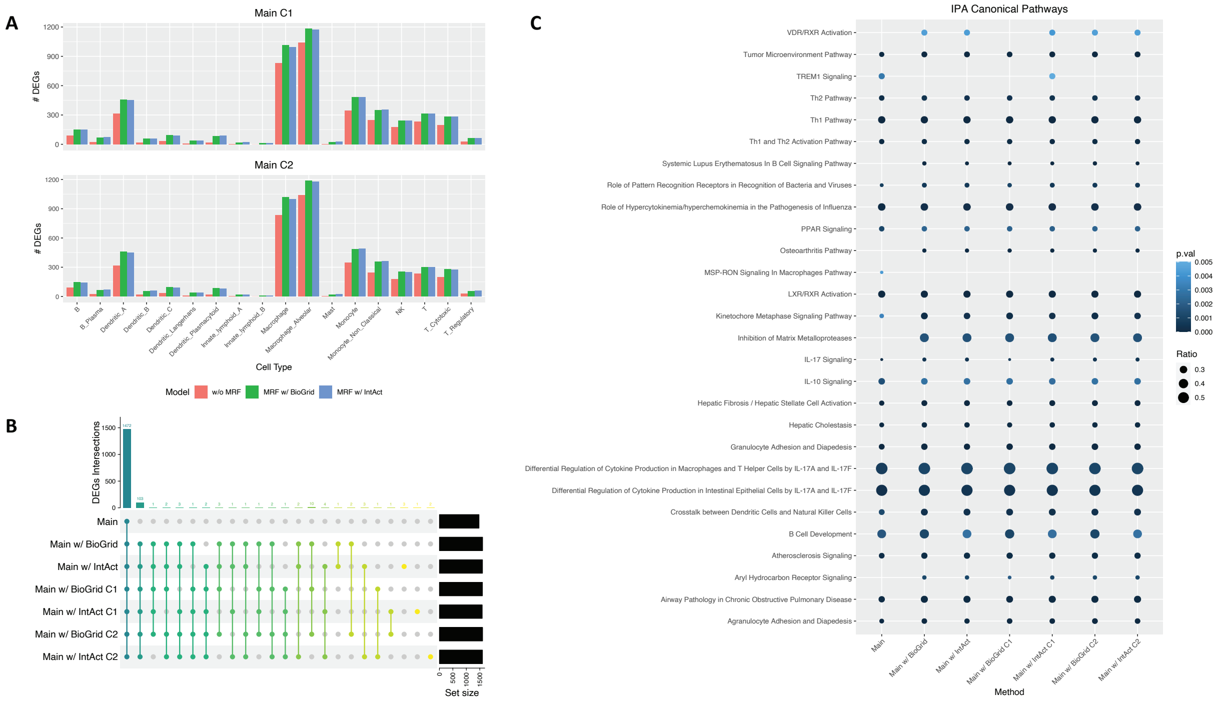

Supplementary Figure 6. Results from IPF scRNA-seq data analysis. **(A)** Number of cell-type specific DE genes identified using two-sample t-test statistics alone, and two MRF models with the BioGrid and IntAct database and cell networks C1 and C2. **(B)** The UpSet plot shows the overlap of DEGs identified by the Main model, two Main MRF models, and four Main MRF models with C1 and C2 cell network structures. **(C)** The top canonical pathways from Ingenuity Pathways Analysis. The p-value cutoff was set at 0.005 and gene ratio cutoff was set at 0.2 in order to better visualize top enriched pathways.

Supplementary Table 2. Number of DEGs with Reduced Number of IPF Patients

Method	Full Dataset	Reduced Dataset	Overlap
Main (t-test)	1472	1188 – 1266	1149 – 1227 (96.7% - 96.9%)
Wilcoxon	1562	1318 – 1400	1298 – 1367 (96.4% - 99.0%)
MAST	1721	1561 – 1638	1549 – 1617 (98.7% - 99.5%)
NegBinom	1595	1381 – 1432	1360 – 1408 (97.1% - 98.5%)

* The ranges given in the last two columns were based on five reduced data sets with random samplings.

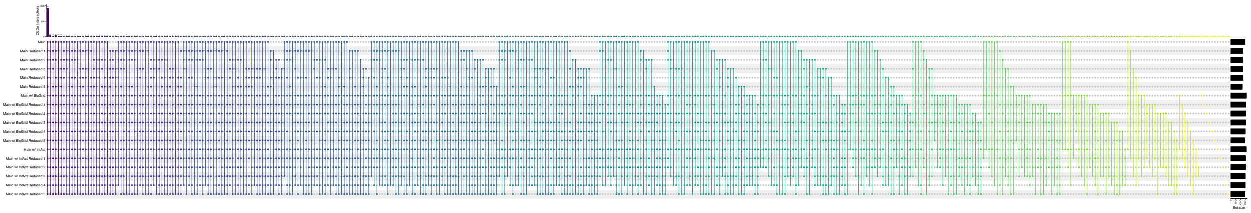

Supplementary Figure 7. The UpSet plot shows the overlap of DEGs identified by the 3 Main models with the full data set and 15 corresponding models with the reduced data set by random sampling of five times.
